# Incidence and investigation of Covid-19 trend in Babol, northern Iran: A Joinpoint regression analysis

**DOI:** 10.22088/cjim.13.0.236

**Published:** 2022

**Authors:** Sara Khaleghi, Hossein-Ali Nikbakht, Sajad Khodabandelu, Soraya Khafri

**Affiliations:** 1 Student Research Committee, Babol University of Medical Sciences, Babol, Iran; 2 Social Determinants of Health Research Center, Health Research Institute, Babol University of Medical Sciences, Babol, Iran

**Keywords:** Trend, Joinpoint, Pandemic, Incidence, COVID-19

## Abstract

**Background:**

In December 2019, China released the first report of the coronavirus (COVID-19). On March 11, 2020 the World Health Organization (WHO) characterized the COVID-19 as “pandemic”. The rapid occurrence of positive cases motivated this study to examine the trend of incidence cases.

**Methods:**

We used the data from the database of the Deputy of Health of Babol City and in Iran, the country report of definite cases of the disease that was reported to the World Health Organization had been used. This study was a cross-sectional study and the data from period of 56 weeks (from February 24, 2020 to March 20, 2021) were gathered. Descriptive analysis with SPSS20 and data classification with EXCEL2016 and Joinpoint regression with Joinpoint trend analysis software 4.9.0.0 identify the significant changes in the temporal trends of the outbreak.

**Results:**

In this study, 11341 patients with a mean age of 53.56 years, of whom 5865(51.5%) were males, were studied. Three waves of Covid19 were created. AWPC (average weekly percentage change) incidence rate with a slope of 2.7 was estimated for Babol and 6.2 for Iran. The incidence was higher in men in the first wave of 1887(55.6%) and so is the third 2373(50.1%), the average age in the third wave (50.92) was lower than the other waves as well.

**Conclusion:**

The incidence of coronavirus in men was higher in three waves and also the incidence was increasing in younger age groups. Also, due to the observance of health protocols and quarantine during the peak in Iran and Babol, we witnessed a decrease in incidence.

In December 2019, China released the first report of the coronavirus (1). About a month later, the World Health Organization (WHO) declared a state of general emergency, and in March 2020, upgraded the main disease to be epidemic. At the end of June 2020, data showed that more than 10 million people worldwide were infected with coronavirus (SARS-CoV-2) due to acute respiratory syndrome, and more than 500,000 people died (2). The disease spread rapidly in all provinces of Iran within 15 days (3). Non-pharmacological interventions such as early quarantine, travel bans or movement management, and restrictions in public communities, as marketing activities to prevent or transmit COVID-19 to "uniquely flatten" the prevalence in China and other global hazards were reported (4-7). Governments around the world have taken steps to prevent the transmission of the coronavirus because COVID-19 has caused economic disruption beyond the global health crisis, and its impact on the economic system is being felt (8).

These data provide a good opportunity for researchers and government officials to understand the prevalence of the disease better and predict disease patterns as well as provide technical support through data analysis. In addition, the data provide a better understanding of how the corona virus spreads temporally. To assess whether the trends changed over time, we used the Joinpoint segmented regression method. The Joinpoint regression model was used to study the time-varying trend because the basic assumption of Joinpoint regression is that the data flow is not the same during the period And the goal is to provide a model that summarizes the trend of coronavirus incidence (9). Joinpoint regression is very useful in various fields such as sample time series data change especially in health studies (10). 

Towards stability and decline therefore, the purpose of this study is to describe the behavior of the historical collection of Covid-19 cases and comparisons in the city of Babol and Iran and identify changes in trends during the epidemic period. Joinpoint software is also used to identify trends and identify the epidemic Joinpoint, as well as calculate trends on days sensitive to increasing or decreasing trends, the process is calculated to obtain more information and compare. The aim of this study was to provide a model that fits the time series data and evaluate the Joinpoints (which in this study are defined as when the accumulation rate of incidence changes from increase to decrease or vice versa). This study was also conducted to identify the temporal patterns of epidemic in the city and analyze the changing patterns of infection by the Joinpoint regression model. This statistical model identifies significant changes in a pattern over a period of time by assuming a trend between inflection points (“Joinpoints”). A significant change between one point and the next is assumed to mark an inflection point and the beginning of a new regression trend. One of the advantages of this method is the ability to identify the number and location of changes in the trend, and to estimate the average weekly percent change (AWPC) for each period defined between inflection points. Joinpoint regression models are particularly useful in evaluating continuity constraint at change points over time. 

## Methods

Study population: In this population-based study, time series data from the database of the Deputy of Health of Babol City were collected. These data were analyzed separately based on age, sex, incidence and duration. Also, for Iran data, the country report of definite cases of the disease that was reported to the World Health Organization was used. To reflect the whole trend, data were systematically collected from February 24, 2020 to the end of March 20, 2021, which included 3 waves of epidemics.

**Statistical model: **In this study, we examined the number of new cases of COVID-19 on a weekly basis based on gender, age groups and the number of cases in the population of Babol. "Joinpoint regression model" is a technique for examining important points and the number of changes over a long period of time for population and health trend. The term "Joinpoint" describes and shows us when the change began. This model uses data over a long period of time because we can have a clearer pattern. Also in this model, p-values <0.05 are considered significant. Based on the incidence of cases confirmed through this software, the Joinpoint time between the two stages, the growth rate of the first stage (slow growth) and second stage (fast growth) can be identified. To show changing patterns of the epidemic. The Joinpoint regression model is fundamentally different from the conventional regression model. In the Joinpoint regression model, the purpose was to identify the connection points (s) and estimate their location in the model and was not adjusted arbitrarily. The minimum and maximum number of Joinpoints are predetermined but the final number of Joinpoints or their place and time is determined when the trend change is statistically significant. This model first identifies the time of the Joinpoint, when there is a change in trend. It then calculates the percentage change in time, which represents the amount of change between the two Joinpoints And when the number of Joinpoints reaches zero, the model is reduced to a simple linear regression model(9) .

In this method of Joinpoint regression, for each piece we will have a Fi (X) linear regression function with different parameters. The regression model of Joinpoints for r fragment is generally:

fx=EY|X=f1x:β1 x≤τ1f2x:β2 τ1≤x≤τ2 :frx:βr τr-1<x

Which τi represents the Joinpoints and fi (X, β_i_) of the regression functions for each component. Items that should be considered in the Joinpoint regression include the number of Joinpoints, the location of the Joinpoints, and whether the breakpoints are known or unknown. The Joinpoint regression model is defined as follows:

E[y |x] = β_0_ +β_1_ x +δ_1_ (x –τ_1_ )^+^+…+ δ_k_ (x –τ_k_ )^+^

Where k is the number of unknown change points, τ_i_ for i = (1,…, k) are the places of unknown change points and δ_i_ are the regression coefficients of the parts(11). This model estimates the weekly percentage change (WPC) and the average weekly percentage changes (AWPC) to identify Joinpoints where significant changes have occurred during the epidemic period. A logarithmic transformation of COVID-19 expression was applied and the option of heteroscedastic errors were considered as standard errors, which due to heterogeneity and variance of Poisson assumptions were used. We also estimated statistical significance (p<0.05 with 95% confidence Interval) for each WPC and AWPC estimate (12). For statistically significant fits, WPC can be used as an "increase" or "decrease" trend. Models with zero to three connection points were analyzed here and the models that best fit the observed data were selected. A comparative analysis was performed between the period before and after the start of quarantine restrictions. Also, descriptive analysis using SPSS software Version 20 was used to check the frequency and indicators of descriptive statistics and EXCEL software Version 2016 was used to classify the data. Also, all statistical analysis and time trends in the incidence of COVID-19 were performed using Joinpoint trend analysis software Version 4.9.0.0 (US National Cancer Institute). 

## Results

According to our results, of the 11,341 people affected, 5,865 (51.5%) were males and 5,476 (48.1%) were females. Percentage and frequency of age groups are reported in table 1 and descriptive information for three epidemic waves based on age groups is reported in table 2. The results showed that the highest frequency was observed in the age group of 70-61, 60-51, < 30 and. respectively in table 1. Also, the highest mean age was observed in the first wave and the lowest in the third wave in table 2, and the mean of the general age group in the three epidemic waves was 53.56. Number and percentage of men in the three waves of the epidemic, respectively 1887 (55.6%), 1605 (50%) and 2373 (50.1%). 1504 (44.3%), 1604 (50%) and 2368 (49.9%) were observed for women, respectively. 

Based on the epidemic waves generated in the Joinpoint model, a descriptive analysis was performed as follows:

**Table 1:** Frequency of age group for three waves of epidemics in the joinpoint model

** Age group**	**N (%)**	**Epidemic waves**		
		**one** **Frequency (%)**	**two** **Frequency (%)**	**Three** **Frequency (%)**
<30	1703 (15)	271 (7.9)	479 (14.6)	978 (20.3)
31-40	1238 (10.9)	395 (11.6)	385 (11.8 )	469 (9.7)
41-50	1471 (12.9)	520 (15.2)	412 (12.6)	552 (11.4)
51-60	1986 (17.4)	672 (19.7)	572 (17.5)	763 (15.8)
61-70	2059 (18.1)	659 (19.3)	584 (17.8)	833 (17.3)
71-80	1657 (14.6)	534 (15.6)	443 (13.5)	700 (14.5)
>80	1227 (10.8)	359 (10.5)	362 (11.1)	525 (10.9)
total	11341 (99.6)	3410 (99.8)	3237 (98.9)	4822 (100)

**Table 2:** Descriptive analysis of age group for three epidemic waves in the joinpoint model

**Epidemic waves**	**Mean**	**SD**	**Median**	**Interquartile Range**	**Min**	**Max**
one	57.12	18.65	59	26	1	99
two	53.54	22.48	57	31	0	99
three	50.92	25.74	56	35	0	101
total	53.56	23.02	57	31	0	101

**Figure 1:**
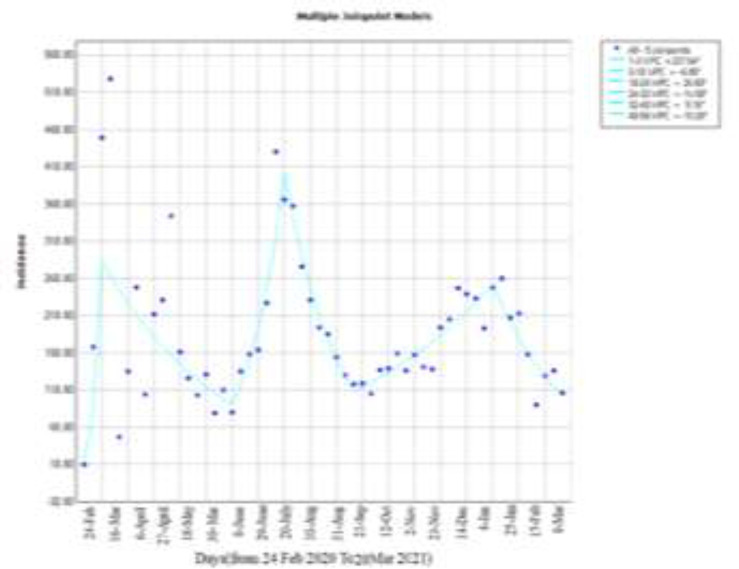
incidence trend until 20 march 2021 in babol city

Joinpoint regression in this study has 2 approaches: Once we performed the analysis in general and once with the subgroup analysis. In these graphs, we plotted the number of weekly occurrences against time. Points are real data, and connection lines represent regression lines drawn by Joinpoint software. To the right of each figure is the time period for finding Joinpoints and the period percent changes by the program and each time period is connected by a line, which shows its slope. However, not all ranges are always statistically significant, and the best model with a star in the figure is shown as the best model. According to figure (1), during a period of 56 weeks in the city of Babol, five Joinpoints were observed for incidence cases. The Joinpoint and trend change in figure (1) are observed in the third, eighteenth, twenty-fourth, thirty-second and fiftieth weeks, indicating three epidemic waves in this model. The study of spatial-temporal patterns can help us understand the mechanisms of disease spread in a population, allowing the detection of behaviors in time that represent characteristics related to dissemination

The use of segmented regression analysis enabled us to identify points in time when the trends changed significantly, generating quantitative hypotheses about changes in the behavior of the pandemic in Babol City and Iran. In this study for Babol City, AWPC incidence rate with a slope of 2.7 and confidence interval (-2.5, 8.1) was estimated also for Iran AWPC incidence rate with a slope of 6.2 and confidence interval (2.3, 10.2) was estimated.

**Figure 2:**
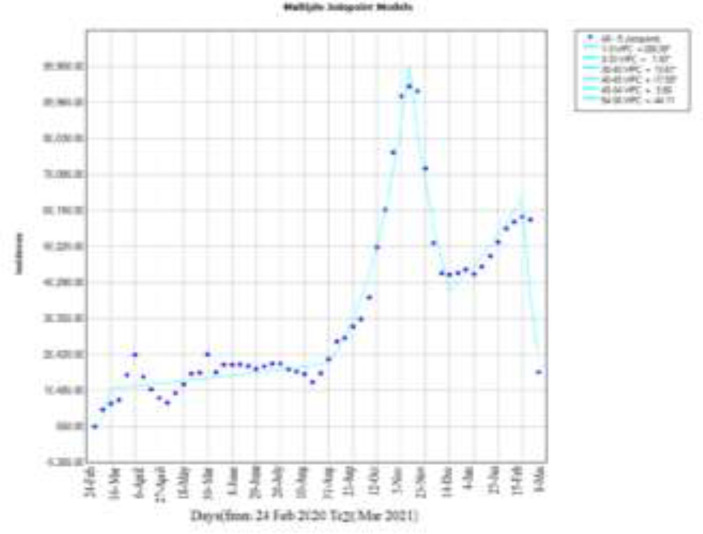
incidence trend until 20 march2021 in Iran

 In these models, the (WPC) weekly percentage change in the incidence rate for positive cases indicates an increase in incidence cases. And in negative cases, we see a decrease in incidence and you can see it in figures 1&2.

At this stage, we intend to examine the patterns of Joinpoints by gender and age groups, and to examine the incidence and trend separately.

**Figure 3:**
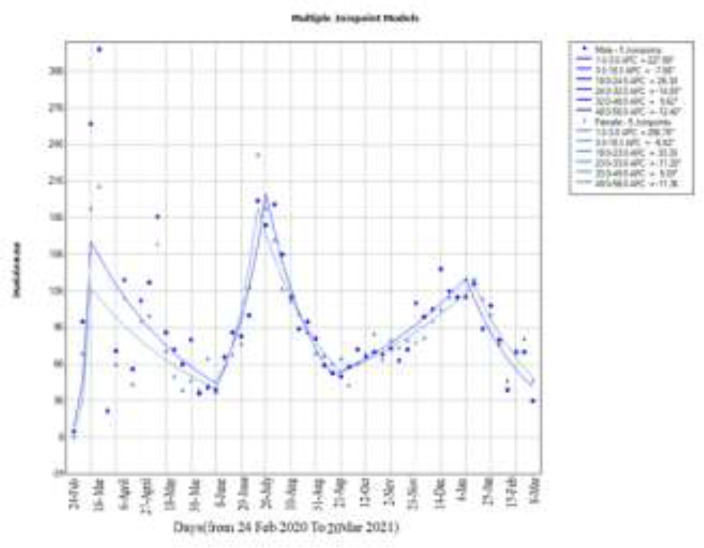
incidence trend until March 20 by sex in Babol City

The regression model of Joinpoints in the two models is as follows: In this figure, five Joinpoints were created for males and females and you can see it in figure 3. The WPC for men and women is created in the form of 6 lines and is displayed in the same figure. The AWPC in this model for the male gender was estimated to be 2.1 with a confidence interval (-3.4, 7.9) and for the female gender was 3.2 with confidence interval (-1.9, 8.5). 

**Figure 4:**
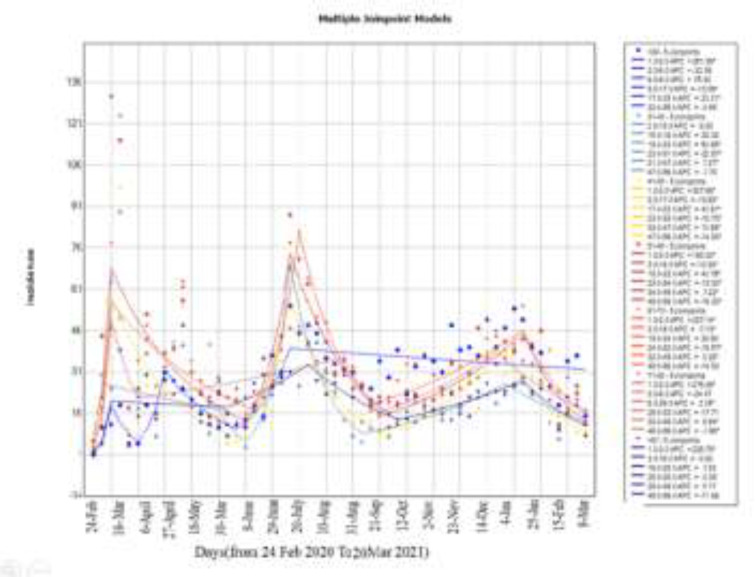
Incidence trend until March 20 by age in Babol City

 In figure 4, for age group in this one-year AWPC and the confidence interval for the first to seventh age groups, respectively : 5.8 (-1.4, 13.5) and -1.3 (-10.1, 8.2) and 1.8 (-4.4, 8.4) and 0.9 (-4.6, 6.6) and 2 (-4,3, 8.7) and 2.9 (-3.4, 9.7) and 3.4 (-1.9, 9) were estimated. This indicates that the estimated incidence during this one-year period was average with this slope ascending or descending

## Discussion

In Babol City model in which the incidence cases were examined in general, in 9 March 9, August 3, February 1, 2021, with the most incidence cases that had an upward slope and as a result, Joinpoint was created and in the weeks of June 22, June 28 September, and on March 15 , 2021 we saw the lowest incidence, which indicated a downward slope and thus the creation of Joinpoint. In this model, AWPC was estimated with a slope of 2.7, this means that on average, the estimated incidence during weekly intervals with a slope of 2.7 will take an upward trend. In other words, AWPC shows that a single number can be used to describe the average of WPCs over a period of time. It shows that the estimated incidence during this period was average with this slope ascending or descending. AWPC shows that a single number can be used to describe the average of WPCs over a period of time. WPC and AWPC can also be used as a suitable measure to explain and summarize the process. Here AWPC = 2.7 reports that during this year we are witnessing an increasing trend and each week compared to the previous week, we will have an average of 2.7% increase in the trend in the city of Babol and in the future, under constant conditions, if no intervention is made with the same trend, every week we will see an average increase of 2.7%. Figure 2 showed AWPC = 6.2 in the Iranian data model and AWPC = 2.7 in the city of Babol. Based on these results, it can be said that we have witnessed an increase of 6.2% of cases in Iran every week compared to the previous week. But in Babol compared to Iran with a lower slope but still up 2.7% every week compared to the previous week we have seen an increase in cases comparing with the graphs, it can be said that in Iran and Babol from the beginning of February 24, 2020 to March 9, 2020, a mutation was observed that is due to an increase in incidence and in Babol, after this leap, we have witnessed a decrease in incidence and in Iran, with a very small upward slope, there was an increase in cases Which could be the result of global quarantine on this date and increased compliance with health protocols. We have witnessed an upward jump in the city of Babol from June 22, 2020 to August 3, 2020. This leap occurred shortly after September 14, 2020 to November 23, 2020 .We have witnessed a very sharp increase in cases in Iran and a decrease in incidence, in which this reduction has been the result of quarantine and closure and more compliance with health protocols. Moreover, from September 28, 2020 to June 18, 2021, we have witnessed another upward jump, which was observed in Iran from December 28, 2020 to March 1, 2021. 

The second model was drawn by gender. The highest frequency was observed in the first and the second waves for male gender and in the third wave, the frequency was almost equal, which indicates that during these three waves, men were affected more and earlier. In this model, AWPC and confidence interval for males and females were observed with a slope of 2.1and 3.2, respectively. This means that on average, the estimated incidence during weekly intervals with these slopes will be upward. Here the AWPC reports 3.2 for females and 2.1 for males, which over the past year has seen an upward trend and every week, compared to the previous week, we will see an average increase of 3.2% and 2.1% in Babol. AWPC was also observed and in the future, under constant conditions, if no intervention is made with the same trend, we will see an average increase of 3.2% and 2.1% each week. 

As can be seen, the highest increase in incidence was seen based on the average weekly percentage changes in female gender. It is expected that if the intervention is not done and the conditions are stable, we will see an increase in the incidence trend in women in Babol with an average of 3.2%. Furthermore, in men with a slope of less than 2.1%, we see an increase in incidence.

And according to the Joinpoint chart in three waves, it can be seen that the rate of corona incidence in men and women increased and decreased together and only in the first wave in the third week when the first breaking point was observed. The incidence was much higher in men and in the second wave at the Joinpoint 23 cases of incidence corona is one week ahead of men in women. But in other cases, the incidence has decreased and increased almost gradually. In general, men are at risk during these three waves due to reasons as being more social, immunological, lifestyle and habits such as smoking, self-care or other factors (13, 14).

The third model was divided into age groups. The mean age in the third wave (50.92) was lower than the other two waves Also, the highest frequency was observed in the first wave in the age group of 51-60 years, the second wave in the age group of 61-70 years and in the third wave, the highest frequency was observed in the age group <30 This indicates that the virus is affecting the lower age group in the third wave, and corona is spreading among young people. As can be seen, the highest increase in incidence was seen based on the average weekly percentage changes in the age group <30 with AWPC = 5.8. It is expected that if the intervention is not done and the conditions are stable, we will see an increase of 5.8% in the incidence trend in Babol in the age group <30 every week.

 In general, the highest incidence during this period was observed first in the age group of 61-70 (18.1%), then followed by the second group in the age range of 51-60 (17.4%) and finally the third group in the age range of < 30 (15 %). This result can be seen in the Joinpoint diagram for age group (figure 3), which has the highest incidence for age groups 4 and 5 and age group 1. As of from the 23rd week, it has experienced the most cases of uniformity and according to the results of Joinpoint regression model such as weekly percentage changes (wpc) and average weekly percentage changes (AWPC), it can be said that on average, the increase in incidence was faster in times when corona constraints were not applied. However, reaching a Joinpoint for an epidemic does not necessarily mean the end of the epidemic and the reduction of incidence in general because SARS-COV secondary transmission continues in China and around the world Therefore, we still need to make more efforts to reduce the incidence. The first wave of the epidemic began when no standard and comprehensive protocol for the treatment of patients was published worldwide. The scientific evidence was largely the result of limited study experience, usually in small sample-sized populations or small study areas that offered different treatments to health care professionals(15). In the beginning of this study, the number of newly identified cases increased abruptly because at that time, extensive RT-PCR methods for accurate diagnosis of COVID-19 in symptomatic patients were not readily available. Thus, fewer patients were examined using RT-PCR during the first wave of the disease Another factor to note is that during the first wave, more patients with COVID-19 needed respiratory support(16, 17). This sudden increase was mainly due to non-compliance with health protocols as well as the increase in testing centers to facilitate early treatment and care for suspected cases and to prevent secondary transmission Also, restrictions and quarantine can be one of the factors in reducing the number of cases. In addition, age, sex, disease severity, and comorbidities are the other important factors that can explain the difference in mortality observed during these three waves (18-20) .

This study also has limitations, first we did not have accurate information about each patient, information such as underlying diseases and living conditions, etc., and this prevented accurate assessment of possible triggers for the epidemic. Second, a number of patients with corona have not been referred to a health center for testing Therefore, our analysis may not show the exact status of the epidemic. However, this study still provides us with important information that can be useful as a solution to the reaction to this disease. Further research could be done on the effect of vaccination on the incidence and on the incidence of recurrence and mortality. The present study aims to evaluate the impact of quarantine and isolation strategies in this region. Despite many efforts to prevent it, the burden of SARS-CoV-2 prevention and control is still very high in this area thus, even after complete elimination of the infection, preventive measures are still needed to minimize the possibility of future relapses.

In this survey, it was discovered that the prevalence of Covid 19 was growing such that men had a larger rise than women. The age group decreased from the first to the final wave, therefore the maximum incidence in the third wave was shifted to the age group <30. In this age group, this problem warns the necessity to restrict, monitor social distance, use masks, and adhere to additional health protocols. The biggest leap in Iran and the city of Babol was the second leap and many people lost their lives in this history. Then, according to the decision of the Ministry of Health, the quarantine and the general closure and the order to observe the health protocols were given, and this issue reduced the incidence and control of the situation. This information can be useful for medical emergencies to rescue patients as well as predict mortality over a specific period of time and even after complete elimination of the infection, preventive measures are needed to minimize the possibility of a future relapse. In addition, public health officials should investigate the factors that cause the rapid and persistent transmission of Covid 19 disease in these areas.
